# Editorial Notes

**Published:** 1889-01

**Authors:** 


### HOMŒOPATHIC PHYSICIAN,

A MONTHLY JOURNAL OF

HOMŒOPATHIC MATERIA MEDICA AND CLINICAL MEDICINE.

"If our school ever give up the strict inductive method of Hahnemann, we are lost, and deserve only to be mentioned as a caricature in the history of medicine."—CONSTANTINE HERING.

Vol. IX. JANUARY, 1889. No. 1.

### EDITORIAL NOTES.

A ONE-SIDED VIEW.—We have always held the opinion that those who lay so much stress upon the curative powers of the so-called Tissue Remedies, to the exclusion of other remedies, have a very one-sided view of the proper uses of drugs, as they should be prescribed under the Law of the Similars. Our conception of drugs used in homœopathic practice is that each drug has an individual character to which no other drug is exactly similar; therefore, one drug can never be said to take the place of another drug. If one drug be indicated in any case of sickness, it is proof positive that no other drug will do for that case; another drug may cover the symptoms very closely but not perfectly. It is close attention to this accuracy in adapting drugs to individual cases that marks the dividing line between the homœopath and the non-homœopath; it also separates success in practice from failure. This one-sided view of the uses of homœopathic remedies is caused by a false pathological teaching, which would have us prescribe for diseases rather than for persons; according to this view, we have certain remedies which are good for "fever," others for "rheumatism," etc. No teaching could be further from true Hahnemannian philosophy than this; and none is the cause of more eclectic practice.

To show how far from true homœopathic philosophy these views lead one, we quote from a letter recently received from a physician, who seems to place the utmost confidence in the curative powers of these "tissue remedies." He writes:

"I have been using the tissue remedies for about five years, and have found them sufficient in almost all cases (perhaps in all, if I could always see the indications), and have used the old remedies when I could not cure with tissue remedies. If these twelve remedies cover the majority of cases (almost, if not quite all), why carry two or three hundred? Some cases may be so complicated as to require alternation or combination, and in that case the indicated vegetable remedy may be selected, if desired or preferred, which contains such combination as is needed. But where we can so prescribe, I think it better to use the single (uncombined) remedy; for this reason I prefer the direct medication of Schüssler. I find Ferrum phosphoricum, for instance, takes the place in all inflammatory troubles of Aconite, Gelsemium, and Veratrum viride; why, then, carry these three additional drugs, when the Ferrum-phos. does the work as effectually as either or all of them? I find Kali phosphoricum covers the chief symptoms of Baptisia, Pulsatilla, Cimicifuga, etc.; why not use the one and not lumber the pocket with a half-dozen extra remedies of the same nature? I do not say throw away the old remedies, but study the new well and prove them until we get them perfected, and supply the deficiency with the old."

The opinions of this physician give us a very fair example of this one-sided view of drugs; Aconite, Gelsemium, and Veratrum viride are viewed only as antiphlogistic remedies, of their wider and much more useful sphere of action nothing seems to be known. These drugs are only indicated in their peculiar kinds of fever or inflammation, not by any means in every case of such disease. Each has its peculiar characteristics which indicate the cases for which it is useful. These drugs are no more interchangeable than are any three men.

The symptoms which call for the exhibition of these three drugs are so totally unlike that one cannot imagine how any physician could expect to cover the three by any one other drug. Indeed, there is no one drug in the materia medica that can fill the peculiar sphere of Aconite alone; and as Gelsemium differs so greatly from Aconite, the problem becomes more difficult; but when Veratrum viride is added to the problem, it becomes simply a *reductio ad absurdum.*

No sentence was ever penned in homœopathic literature which has done so much harm as the one which called "Aconite the homœopathic lancet." Aconite is not a febrifuge; it is only occasionally indicated in fevers, and only then when its peculiar mental and nervous symptoms are present. Let us study our materia medica in its broadest sense; let us not contract, but widen the range of action of each remedy, until finally we shall have a remedy for every possible combination of symptoms.

---

The Dual Action of Drugs.—It is often said that a rose would smell just as sweet under any other name, and so it is with drugs; they will act just as well under the name of Dual Action as under the undisguised title of Similia. The allopaths have found this out, and are using small doses of drugs to cure symptoms which are caused by larger doses of the same drug. Hahnemann declared that drugs would cure in the sick such symptoms as they were able to produce upon the healthy; the allopaths declare that "small doses of medicines, administered to antagonize pathological conditions such as could be caused by toxic doses of the same drug," are very efficacious. These two declarations sound suspiciously alike, and give the impression that the latter one is but a crude theft of the former! In the following quotation, it will be observed that Dr. Reed denies that this "dual action" is homœopathic; he expressly declares it to be "antagonistic" (whatever that may mean) in its action. But how can small doses cure and large doses cause the same pathological conditions, both acting antagonistically?

We quote a part of Dr Reed's views—they are interesting as showing the tendency of a part of old-school physicians toward Homœopathy. Dr. Boardman Reed, of Atlantic City, N. J., in a recent controversy with Dr. Horatio Wood upon the double action of medicines, as illustrated by Digitalis, has rather the better of the argument. The editor of the *Therapeutic Gazette* (Dr. Wood) made a very lame attempt to prove that he was right, and Dr. Reed wrong, in his own publication.

The authorities cited, and the powerful reasoning put forth by Dr. Reed, give the self-supposed infallible therapeutist (Dr. Wood) the worst of the argument. Dr. Reed has further strengthened the position he originally took by an able communication in the *Medical and Surgical Reporter*, November 24th, which he concludes as follows:

"The editors of the *Gazette* offer to award me great glory if I will 'prove in the laboratory' that Digitalis can paralyze heart muscle, and also prove, in the same way, the contrary of various propositions which they lay down. My answer is: 1st. That I am not writing up this matter for glory or credit, but for other reasons, as already explained. 2d. That other men, who are experts in laboratory work, have demonstrated what is demanded regarding Digitalis, and have furnished materials, as a result of their experiments, which prove, in my opinion, that all the drugs referred to have double actions.

"If the aforesaid editors cannot be convinced by the testimony of eminent authorities in their own special field, they would not believe even one raised from the dead, still less my own testimony, supposing that I could afford to equip a laboratory and find time from a busy practice to make the necessary experiments. If they really doubt that Aconitine, Veratrine, and Viridine have double actions, and will accept as conclusive the experiments of any reputable pharmacologists, I will undertake to furnish the necessary evidence; and in case it should turn out that any of the drugs mentioned has not been sufficiently studied, I will then gladly make additional experiments, either on animals or on men, or on both, to supply the deficiency. Otherwise, it will be quite useless to prolong this controversy. At some future time, however, I may publish numerous reports of clinical cases, showing the *efficacy of unusually small doses of medicines, administered to antagonize pathological conditions such as could be caused by toxic doses of the same.* I hope then to be able to demonstrate at length and with sufficient clearness to convince even the most timid therapeutist that he need not be deterred by the fear of treading on heretical ground, from curing his patients with the smallest effective doses, whenever these happen to suit best.

"Indeed, one of the most satisfactory things about the theory of the double action of medicines is that it affords an all-sufficient scientific basis for maintaining that the small-dose effect, as well as the large-dose effect, is really antagonistic to the disease. It thus quite does away with the necessity for lugging in the irrational dogma of *similia similibus curantur* to account for such cures as those of vomiting by drop-doses of wine of Ipecacuanha, or of Fowler's solution, and of diarrhœa by fractional parts of a grain of gray powder, Rhubarb, or Podophyllin. In short, it affords an intelligible and rational explanation of all curative effects obtained with relatively small doses of tissue-disturbing remedies, whether administered by regular physicians or by homœopaths, when the latter do not administer amounts so infinitesimally small as to be incapable of producing any effects."—*Medical Register.*

